# Opinions regarding refractive surgery today in Romania

**DOI:** 10.22336/rjo.2017.15

**Published:** 2017

**Authors:** Filip Mircea

**Affiliations:** * Amaoptimex, Bucharest

Eyeglasses are medical devices whose purpose is to improve the sight deficiency of the eyes with diopters. The reaction of the patients who find out that they have this need, at their first medical consultation, can be different: some of them accept it straightforward, some are more or less upset, and others cannot conceive this option. The patients from the last category look for a way to get out of this so-called “nightmare” because, for most of them, life without glasses is a strong desire.

The options are different, starting with not wearing glasses, the result being an unsatisfying compromise, which can have long-term consequences, starting from amblyopia to the impossibility/ difficulty of performing a specified task/ job (e.g. driving). Other options are contact lenses and refractive surgery by laser or other techniques.

Refractive surgery implies specific conditions that need to be evaluated by the doctor. Myopia (most frequently), hyperopia, and astigmatism can be treated through this type of surgery.

Generally, the current laser techniques used are PRK, Lasek, Lasik, SMILE.

The most frequently used technique worldwide is Lasik, and, in Romania, both Lasik and PRK.

ICL (lens implant in front of the crystalline lens) can be used in patients with high myopia or hyperopia.

Patients who require refractive surgery most often have myopia. Regarding the conditions they have to accomplish, the diopters must be stable for at least one year before surgery and the patients must be at least 21 years old at the time of the surgery. As far as the age at the time of the surgery is concerned, people want to be perfect between the ages of 20 and 40 and, of course, this is the age group with the highest addressability.

Considering the indications, myopia up to 6 diopters, astigmatism up to 3 diopters and hyperopia up to 1.5 diopters can be corrected with PRK technique.

The interval can be extended up to 8 diopters for myopia, to 4.5 diopters for hyperopia and to 4 diopters for astigmatism with Lasik.

SMILE addresses only myopia, with or without astigmatism, and this technique extends the interval up to 10 diopters and even 15 diopters if astigmatism is added. The maximum age recommended for this procedure is 40-45 years.

Regardless the technique used for myopia correction, the patient must be advised about the problems he will encounter at the age of presbyopia.

Another goal that ophthalmologists are aiming is that of not wearing glasses either in presbyopia, meaning after the age of 40, nor in high hyperopia, in the onset of accommodative asthenopia. In this case, multifocal IOL implants are the most frequently used solution. The main question that arises is regarding the age. In a study that used well-known Romanian and foreign surgeons, the recommended age for this type of surgery was between 35 and 60 years. Personally, I choose the lower limit of this interval. What can we offer to a young patient aged 27 or 30, with a myopia or hyperopia impossible to treat with laser surgery, who desperately wants not to wear glasses? I believe that these patients can get surgery, too, after a thorough examination and information.

Multifocal IOL implants are used by surgeons in Romania, as well as in other countries, but, in general, in a small percentage of cases.

The results of this procedure are mainly very good.

It takes a careful preoperative examination, as well as a discussion with the patient to understand his needs, but also to disqualify from surgery those patients with unrealistic expectations or jobs not suitable for this technique (e.g. nighttime drivers). 

Usually, a discussion about implanting multifocal IOLs is started after a normal aspect of the macula is seen on tomography. 

The surgical technique has to be very good. What should be mentioned is that the surgeon who uses this type of implants must have access to excimer laser, possibly femtosecond laser to correct a postoperative refractive error, a correction that is usually recommended 6 months after the surgery. What should also be mentioned is that Nd:YAG laser can be used even at 1 month after the surgery.

These procedures are expensive and generally not supported by the insurance companies anywhere in the world.

In my opinion, these multifocal IOLs offer very good results, but they can always be improved and all the producers constantly try to improve the quality of life

**Mircea FILIP** **Amaoptimex, Bucharest**

